# Macon Medical Society

**Published:** 1884-12

**Authors:** 


### Society Reports.

### MACON MEDICAL SOCIETY.

Reported for THE ATLANTA MEDICAL AND SURGICAL JOURNAL.

STATED MEETING, October 7, 1884.

Dr. C. H. Hall, president, in the chair.

The introductory essay was read by Dr. N. G. Gewinner. Subject:

### CEREBRAL CONGESTION COMPLICATED WITH CHOREA, FROM A FRIGHT.

Early on the morning of the 18th of September, I was hurriedly called to see the child of Mr. R., a little boy, aged four years. On my way to the house I learned that on the preceding day the child was playing in the back yard near the fence which divided that yard from the next, when a horse in the adjoining yard suddenly ran up to the fence, and, sticking his head over, neighed shrilly just above the place where the little one sat. This frightened him so much as to nearly throw him into fits, as the mother expressed it, and gave him the jerks, as she called it, for some time after. All during the balance of the day he had been very nervous, and during the night was very restless; would suddenly rouse up with a cry, and throw his arms around its grandmother, with whom he slept. About five o'clock in the morning he began to get cold, lost consciousness, and his face became livid.

I found him lying in a comatose condition, just after a heavy convulsion, his face of a leaden hue, pupils widely dilated, pulse 140, weak and fluttering, temperature 97½°, extremities cold and head very hot; tongue heavily coated, a dark cream color interspersed with bright red papillæ. I at once had the extremities rubbed with mustard, applied bottles of hot water to feet and ice to head, and painted the back of the neck with blistering fluid. Prescribed bromide of potash and bromide of soda, of each five grains, chloral one grain, to be given every hour till relieved. Also gave four grains each of calomel and sugar, and ordered an injection to be given in two hours. After the first dose of the nervine he became conscious and fell into an uneasy sleep. I left him at seven o'clock and returned at nine. Found him still resting, pulse 120, temperature 99½ When I was about to leave, he was seized with what we at first thought to be another convulsion. I soon saw that the movements were not like those of an ordinary convulsion, but rather like those of chorea of the electric variety. Only his left side was affected, and this with jerks of the eye, left corner of mouth, left arm and leg, with the regularity and as often as the pendulum of a clock. His tongue did not appear to be affected, and he was able to swallow without any trouble, and was fully conscious. Continued the bromides and chloral. At 12 M. his pulse had risen to 130, temperature 102 deg., jerking still continuing as regularly as before.

At one o'clock, Dr. W. F. Holt saw him with me. We then prescribed pot. brom. and chloral, of each five grains, to be given every two or three hours till relieved, and directed four grains of quinia sulph. to be given at 12 o'clock at night, and three grains at three o'clock next morning, to ward off the chill which we feared might return. As we were about to leave, the child had a large action of a bilious nature, evidently from the calomel. At six P. M., I found the little fellow sleeping quietly without any jerking. His pulse was still 130, but more regular; temperature 102½ deg. Directed the nervine to be given during the night as he might require, and the quinine at the time specified, if fever was not too high.

On the following morning, September 19, I called at seven o'clock; patient had greatly improved; seven grains of quinine had been given at three A. M. His temperature was 100 deg., pulse 116; no action on the bowels since the preceding day. I ordered a dose of oil to be given at once. This acted nicely, and the patient continued to improve rapidly. He was kept on quinia, bi-sulph., five grains night and morning until the fever ceased, which was about the fifth day. His left arm after the cessation of the spasms was partly paralyzed, and he would lie, if not watched, with his arm doubled under him, apparently without it paining him or his being conscious of it. Friction and the use of a mildly stimulating liniment soon restored the arm to its natural condition.

This case is interesting, first, from its being another example of chorea produced by fright; and secondly, from its being undoubtedly complicated with cerebral congestion from malaria. I am not prepared to say how much the one affected the other. Did the fright hurry on the congestion as well as produce the chorea? The patient was undoubtedly predisposed to chorea, and is of a rheumatic diathesis. I attended him during an attack of infantile convulsions when only three weeks old, and when two years of age I treated him for a slight attack of rheumatism.

In the treatment which was followed, the cold applications to head and heat to the extremities, together with the calomel, appeared to have a happy effect in relieving the congestion, while the chloral and bromide had a like effect upon the chorea. The quinine was used as an anti-periodic to overcome the malaria. The patient is now running about in his usual health. At the suggestion of Dr Holt, I shall place him upon the bromide of iron and arsenic to prevent a return of the chorea.

Dr. Stevens. "Has the child ever had chorea before; and has its health always been good?"

Dr. Gewinner. "Had an attack of convulsions when three months old. General health good."

Dr. Williams. "Chorea, though a perversion of the nervous system primarily, is often due to psychical causes. Fright is the most frequent exciting cause. I have no doubt that any other of the emotions strongly excited may produce the disorder. I remember a case of simple chorea, in a nervous, delicate child, the exciting cause being jealousy. This was the diagnosis of Prof. DaCosta, of Philadelphia, and his treatment was to request the mother to demonstrate more affection towards the child. Under this simple treatment the child recovered in a few days."

Dr. Stevens. "Chorea is dependent on a neurasthenic condition. D gestion and nutrition of body generally may be good, but there exists a perversion of the nervous sensibility. I call the case of a child two years of age in the southwestern portion of the State. This child was very choreic, had no control whatever over the muscles, could not walk, and at length the tongue became affected. I placed her on Fowler's Solution of arsenic and bromide of potassium; entire cure in two months. In early childhood she was very strong, but lost flesh and strength before the choreic symptoms came on. The object of the treatment was to tone up the nervous system, and arsenic is peculiarly adapted to the disease."

Dr. McHatton. "Malarial element was present in this case, with a higher temperature than belongs properly to chorea, and may not the chorea be dependent upon the fever, as convulsions often are?"

Dr. Gewinner. "The child was always anæmic and colorless. Malaria may have brought on the chorea by congestion of the brain.'

Dr. McHatton. "Was there an anæmic murmur at the base of the heart?"

Dr. Gewinner. "Dr. Holt tried to examine for it, but the child was so restless that he failed to get it. The patient was placed on bromide of iron and Fowler's Solution, to be continued at intervals for four or five months."

DISCUSSION OF SCARLET FEVER. The President called upon Dr. Gewinner to give his experience in the present epidemic, as he has treated by far the largest number of cases.

Dr. Gewinner. "Could not get at origin of cases; no traceable contagion. It appears atmospheric. There were three cases in one house. The treatment was mild purgatives and quinine. One case, a boy six or eight years old, temperature 100 degrees on the first day. Tongue not coated, red on edges, no rash. On the second day temperature 102 degrees, sore throat and eruption. Third day temperature 103 degrees. On the sixth day fever and eruption began to decline. The tonsils were swollen during this time with but little ulceration. Treatment, lemonade, flax-seed tea and mild purgatives. The patient had no sequelæ. Desquamation was general, and took place between the second and third weeks. The eruption in all cases was first small, red spots under the skin, beginning on the face and taking in the entire body, at first looking like small measles, and then assumed the regular scarlatina type. All had desquamation and sore throat, with albumen in the urine. One case followed by dropsy and one of stiff neck. The case of dropsy had temperature of 104 degrees during fever. Had eighteen cases in all. I also saw a few cases of sore throat and fever with no eruption."

Dr. C. H. Hall. "In the last forty days have had nine distinct cases. The temperatures have been higher than in those of Dr. Gewinner, from 102 degrees to 104 degrees on the first day. The eruption was distinctly efflorescent, beginning on the neck or back of ears, and lasting about six days. All had more or less throat trouble. One case was sick twenty two days; fever began to decline on the tenth or twelfth day. I think there can be no doubt as to the diagnosis. The eruption usually began on the second day. One case, ten years of age, eruption on second day, lasting six days, desquamation on the twelfth day, tongue red on edges, yellowish white centre, papillæ elevated. I examined three or four cases for albumen, and found traces in only one case. In a family of three children one had a distinct case, the second had headache, lassitude and fever, without any eruption or throat trouble; the third had no distinct eruption, some sore throat and fever; no complications following in these cases. I have used in these cases aconite and belladonna. I use belladonna in all exanthemata. Belladonna is a most remarkable drug, illustrating the similarity of symptoms produced by certain drugs and diseases. Jahrr says, in regard to the physiological effects of belladonna on the skin that "it produces a crawling, itching sensation, a scarlet redness on the face, neck and chest. The tongue becomes red, the papillæ are bright red and inflamed, the throat becomes raw, and there is soreness of the palate, inflammation of the throat and fauces—phlegmanous with violent fever." Wood says, "desquamation of the skin sometimes follows its use." Piffard says, "it produces on the skin a burning and biting sensation; in poisonous doses the whole body is red, dry and hot. It gives rise to a reddish efflorescence, a scarlatina-like eruption." The homeopaths use it in all stages of the disease as long as there is an eruption. I believe in the prophylactic power of the drug—it modifies the disease, and prevents sequelæ; it protects, if not wholly, it does in part. Bartholow and Piffard recommend it for this purpose."

DR. WILLIAMS. "The only case of the disease was a young lady who was attacked with fever and sore throat; in twenty-four hours the rash began to appear on the neck and spread over the body. It was most marked around the neck and in the flexures of the joints. The temperature rose on the second day to 103°. The tongue was at first red at the margins and coated of a yellowish-white over the centre and base; it soon cleaned off, leaving the surface red, the papillæ prominent. On the fifth day the eruption disappeared. A trace of albumen was present in the urine. About 14 days desquamation of a meal bran character began around the mouth, neck and on the hands. No complications remained. The treatment consisted of tincture of aconite, tincture of belladonna and quinine, with inunction of vaseline.

DR. GEWINNER. "Dr. Hall, what size of doses of the tincture of belladonna do you use?"

DR. HALL. "For a child of one year, I use half a drop of the homœopathic mother tincture three times a day."

DR. MOORE. "I have had some cases of continued fever and throat trouble, temperatures ranging from 103.5 deg. to 106 deg, pharynx angry and follicles red and swollen, tenderness outside of the throat. In my own family three of the children were thus affected. I have treated all these cases with calomel and quinine. These cases have had the symptoms described to-night, except the eruption and the desquamation. One case of this fever and sore throat, was followed by swelling and pain in the knee, probably an attack of synovitis.

DR. HALL. "I have had some cases with throat trouble, fever, local pains, etc; but did not class them as scarlet fever. In thirty years' practice in Milledgeville and Macon, I have never seen a case of malignant scarlet fever, never saw a case die except from sequelæ.

DR. FERGUSON. "I have seen four cases which had headache, fever, sore throat, nausea, and vomiting; tongue brown, with red eyes, eruption at end of second day, desquamation in one case. These cases occurred in all parts of town. Was in doubt as to the disease and its cause."

DR. HALL. "I forgot to state that nausea was present in each of the above cases. I regard this as diagnostic."

DR. MCHATTON then exhibited microscopical specimens of the normal kidney, acute desquamation, nephritis, chronic interstitial nephritis, and said: I would like to call attention to a catarrhal nephritis which occurs at the height of the eruption, often free from albumen, characterized by degenerated epithelium cylindroidal casts, with rarely hyaline and epithelial casts. Also in the true parenchymatous nephritis, we get the microscopical evidence of the lesion before the appearance of the albumen in many cases. In cases where albumen has existed and disappeared, we get hyaline casts, cloudy epithelium with a fever, red and white blood corpuscles (not enough to give the albumen reaction by the ordinary tests) proving that inflammatory action exists in portions of the kidney, or in other words, the microscopical evidence of the enphritis lasts about twice as long as the albuminous.

